# Mercury Exposure Assessment and Spatial Distribution in A Ghanaian Small-Scale Gold Mining Community

**DOI:** 10.3390/ijerph120910755

**Published:** 2015-09-01

**Authors:** Mozhgon Rajaee, Rachel N. Long, Elisha P. Renne, Niladri Basu

**Affiliations:** 1 Department of Environmental Health Sciences, School of Public Health, University of Michigan, 1415 Washington Heights, Ann Arbor, MI 48109, USA; E-Mails: mrajae@umich.edu (M.R.); rachlong@umich.edu (R.N.L.); 2 Department of Anthropology, University of Michigan, 101 West Hall, Ann Arbor, MI 48109, USA; E-Mail: erenne@umich.edu; 3 Department of Afroamerican and African Studies, University of Michigan, 4700 Haven Hall, Ann Arbor, MI 48109, USA; 4 Faculty of Agricultural and Environmental Sciences, McGill University, 21, 111 Lakeshore Rd., Ste. Anne de Bellevue, QC H9X 3V9, Canada

**Keywords:** mercury, Ghana, small-scale gold mining, ASGM, GIS

## Abstract

Mercury is utilized worldwide in artisanal and small-scale gold mining (ASGM) and may pose a risk for miners and mining communities. While a number of studies have characterized mercury in ASGM communities, most have focused on a single media and few have taken a holistic approach. Here, a multiple media exposure assessment and cross-sectional study of mercury was conducted in 2010 through 2012 in northeast Ghana with a small-scale gold mining community, Kejetia, a subsistence farming community, Gorogo, and an urban ASGM gold refinery in Bolgatanga. The objective was to assess mercury in a range of human (urine and hair) and ecological (household soil, sediment, fish, and ore) samples to increase understanding of mercury exposure pathways. All participants were interviewed on demographics, occupational and medical histories, and household characteristics. Participants included 90 women of childbearing age and 97 adults from Kejetia and 75 adults from Gorogo. Median total specific gravity-adjusted urinary, hair, and household soil mercury were significantly higher in Kejetia miners (5.18 µg/L, 0.967 µg/g, and 3.77 µg/g, respectively) than Kejetia non-miners (1.18 µg/L, 0.419 µg/g, and 2.00 µg/g, respectively) and Gorogo participants (0.154 µg/L, 0.181 µg/g, and 0.039 µg/g) in 2011. Sediment, fish, and ore Hg concentrations were below guideline values. Median soil mercury from the Bolgatanga refinery was very high (54.6 µg/g). Estimated mean mercury ingestion for Kejetia adults from soil and dust exceeded the U.S. Environmental Protection Agency reference dose (0.3 µg Hg/kg·day) for pica (0.409 µg Hg/kg·day) and geophagy (20.5 µg Hg/kg·day) scenarios. Most participants with elevated urinary and household soil mercury were miners, but some non-miners approached and exceeded guideline values, suggesting a health risk for non-mining residents living within these communities.

## 1. Introduction

Artisanal and small-scale gold mining (ASGM) has recently been identified as the largest contributor to global anthropogenic mercury (Hg) in the atmosphere [1]. Eighty to 100 million people are estimated to depend on ASGM for their livelihoods or be indirectly involved [2,3], and 10 to 15 million people are directly employed by ASGM globally [1,4]. Mercury, a neurotoxicant, is used to isolate gold ore in the ASGM process [5]. There are two forms of Hg: organic and inorganic, which can be elemental (Hg) or inorganic salts (e.g., HgS, HgCl_2_, Hg^+^, Hg^+2^). ASGM utilizes elemental Hg, which poses a risk for human health and environmental contamination as Hg is a potent neurotoxicant [6,7].

Gold deposits are ubiquitous in Ghana and have resulted in ASGM and large-scale gold mining across the country, making Ghana the ninth largest gold-producing country in the world [8]. ASGM has grown tremendously in recent years with the rising price of gold, deregulation of gold mining, chronic unemployment, and increasing poverty [9]. ASGM accounts for 35% of Ghana’s national gold production and employs 500,000 to 1 million people directly or indirectly [8,10]. In the Upper East Region of Ghana, where ASGM has grown rapidly, it is estimated that over 10,000 people are employed directly by ASGM in the Talensi-Nabdam District alone (now separate Talensi and Nabdam Districts) [11].

In Ghanaian ASGM, gold ore is excavated from surface and shallow underground mining, and panning in streams [7]. Ore is generally milled in a grinding machine and screened manually. The fine fraction is mixed with water and gold is concentrated in a wooden sluice box covered with carpets where gold and other heavy minerals are retained. The concentrate is panned with Hg in a rubber pan to form a gold amalgam. The amalgam is heated with a blowtorch to volatize the Hg and leave behind the gold ore with some residual Hg. Large amounts of Hg vapor is deposited locally and can be re-emitted from water and soil surfaces or can be methylated, bioaccumulate, and biomagnify in food chains [1,12]. Surface soils, water bodies, and sediments are the major biospheric sinks for Hg [13].

Exposure to organic Hg, mainly methylmercury (MeHg), is primarily through consuming fish and seafood, although this varies in ASGM communities [14]. Hair Hg concentrations generally reflect MeHg exposures from blood Hg concentrations at the time of hair growth, which grows at an average of one centimeter per month [5,15]. Elemental Hg, used in ASGM, has a half-life of approximately 56–58 days in the whole body and kidneys and can be measured in urine to assess medium-term exposure [5,16]. Urine and hair biological markers are used to assess elemental and MeHg exposures, respectively [5,17].

Soil ingestion is not thought of as a significant pathway of exposure to Hg, but may be of concern if Hg concentrations are high and people ingest significant quantities of soil. In 2010, approximately 400 children were killed and thousands adversely affected from lead poisoning at an ASGM community in northwestern Nigeria, Zamfara [18,19]. In this tragic case, the gold-rich ore also contained lead sulfide (galena), and thus processing the ore lead to extreme contamination of local soil and edible plants with lead. Ingestion of lead-contaminated soils and inhaled dust were determined to be the dominant exposure pathways, while contaminated water and foodstuff consumption were lesser but still notable exposure pathways [19]. The quantity of soil and dust ingested in rural ASGM communities in Ghana are unknown, but there is evidence of the practice of geophagy (habitual, intentional ingestion) in some studies of Ghana and Sub-Saharan Africa, particularly among pregnant women [20]. The species of Hg in soil in ASGM communities is unknown, and may be elemental Hg, which is used in ASGM, or oxidized inorganic or organic species. This practice has been observed among the Ewe in Ghana at a prevalence of 13.9% among adult males (ingestion of 13,000 mg soil/day), and 46.4% among adult females (30,000 mg/day; 150,000 mg/day in upper limit cases) [21], and thought to be an exposure pathway for helminth (roundworms) for women in the Ashanti Region [22]. Fifty percent of pregnant women practiced geophagy in Nigeria [20]. In a northwestern Tanzanian ASGM community, geophagy was prevalent in 45.6% of pregnant women (62,500 mg/day) [23].

An earlier study by our group in Ghana’s Talensi District observed elevated Hg exposure among ASGM miners based on analyses of urine and hair samples [24]. To increase understanding of exposure as well as address continued community concerns, our team conducted a series of follow-up cross sectional studies that were broader in scope to assess Hg exposure in human (urine and hair from ASGM workers and community members) and ecological (household soil, sediment, ore, and fish) samples to provide a more holistic understanding of contamination and exposures. Spurred by the case of lead poisoning in an ASGM community in Nigeria, the work was also utilized to better understand soil Hg contamination (in households situated within an ASGM community as well as in an urban environment near a refinery) as one potential exposure pathway. Spatial methods were used to map key ASGM activities and households, as well as estimate Hg contamination in soil and urinary Hg throughout the ASGM community. We hypothesized that ASGM community participants and ASG miners in particular would have higher hair, urine, and household soil Hg levels than non-ASGM community participants and ASGM community non-miners.

## 2. Materials and Methods

### 2.1. Sampling Strategy and Study Populations

**Figure 1:**
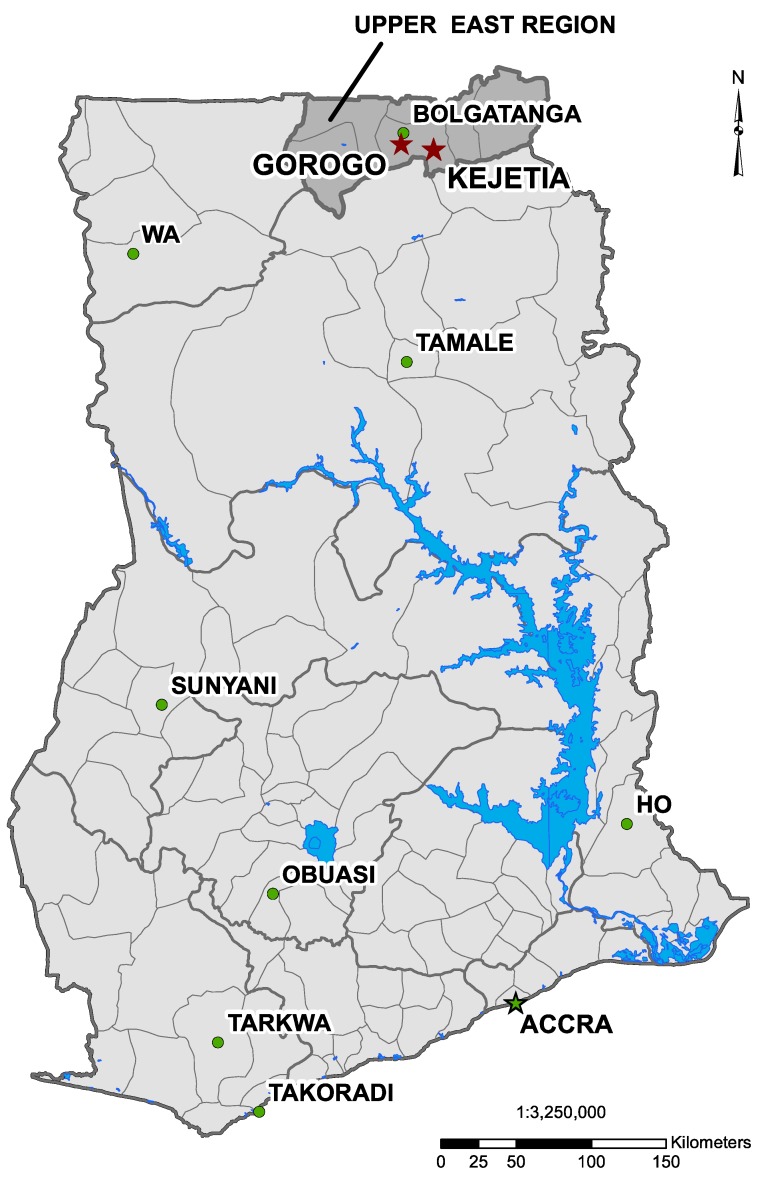
Map of Ghana indicating Kejetia and Gorogo (two red stars), the two research communities, and the municipal capital, Bolgatanga (green circle), in the Upper East Region. (Map produced from data provided by the National Renewable Energy Laboratory [NREL], by Mozhgon Rajaee, June 2012 [26]).

**Figure 2:**
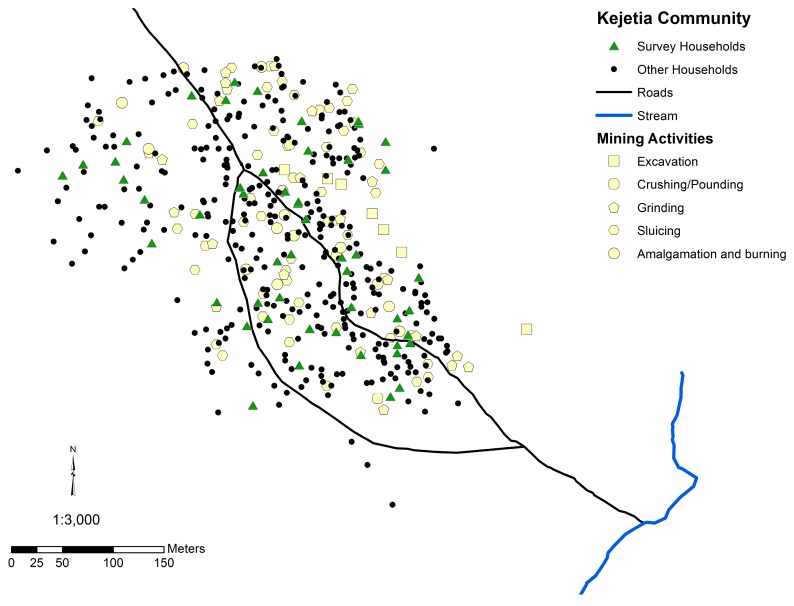
Map of the Kejetia community, indicating households surveyed, community markers, and locations of mining activities.

**Figure S1:**
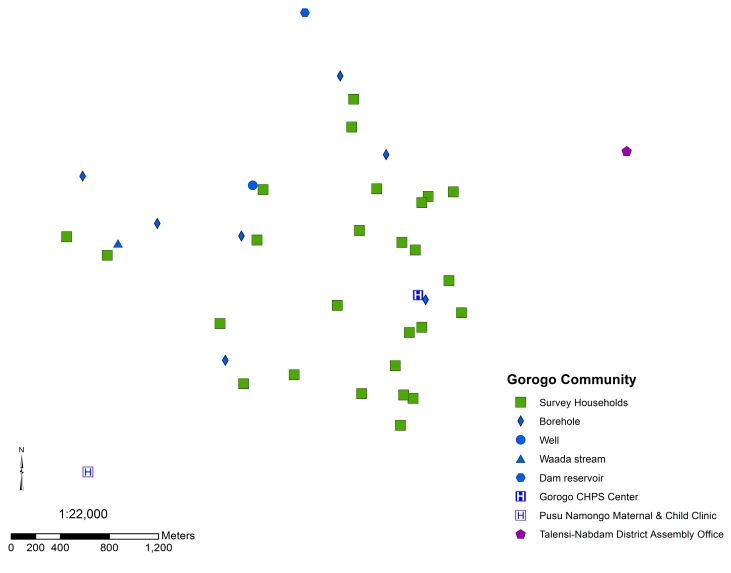
Map of Gorogo households surveyed and community markers.

Data were collected from two communities in the Upper East Region of Ghana, in the prior Talensi-Nabdam District (Figure 1), which has since split into separate Talensi and Nabdam Districts. A small-scale gold mining community, Kejetia (Figure 2), was selected from a prior study of ASGM miners in the area [24]. Kejetia is a community of approximately 2500 people that developed around ASGM in 1995 [11,25]. Gorogo, a nearby, upstream, non-mining community, was selected for comparison (Figure S1). Permission to work with the communities was granted by each community’s traditional chief and assemblyperson in Gorogo, and Institutional Review Board (IRB) approval was obtained through the University of Michigan (HUM00028444).

Participants were recruited to participate in the survey by household. In accordance with cultural norms, households were defined as those who eat from the same “pot”. Lacking community maps and official population estimates, it was impossible to follow true random sampling. In June 2010, women of childbearing age (16 through 49 years) were surveyed from Kejetia through convenience sampling. From May through July 2011, sampling was done in Kejetia and Gorogo. In Kejetia, all households were assigned a set of coordinates using a handheld global positioning system (GPS; Oregon 450; Garmin International, Inc., Olathe, USA). Households were numbered and assigned to a cluster of approximately 20 households based on geographic proximity. Each day, a random number was selected from a bag to identify a household from up to three different clusters for participation. Two to three random households were sampled from each cluster to ensure geographic representation. If a household was not eligible or declined participation, another number from within the cluster was pulled from the bag until an appropriate household was found.

The community of Gorogo was much more geographically dispersed, making clustering infeasible. Households were selected from convenience sampling, by spinning a plastic bottle at the geographic middle of the community and selecting the house pointed to most closely [27]. The bottle was then spun from each participating household to find the next household to be surveyed, and from other geographic locations throughout the community. If a household was not eligible or declined participation, re-spinning the bottle in the same location as the previous spin chose a replacement household.

### 2.2. Surveys

For each household surveyed, a household head and up to three other adults (18 years or older) were interviewed on household characteristics, and their occupational and medical histories. Questions were adapted from the Ghana Demographic and Health Survey (DHS) [28] and the American Thoracic Society Epidemiology Standardization Project [29], with responses being studied in other efforts by our team. English surveys were administered by a team of university students and verbally translated by local Ghanaian translators in the participant’s choice language (Talen, Nabt, Gurune, Twi, Dagbani, English, or Hausa). Translators were trained on appropriate medical terms and health outcomes in local languages prior to conducting the interviews.

The head of household (HOH) or an identified alternative household participant knowledgeable about the individuals in the household completed a survey on demographics of people in the household, household characteristics and amenities, and a 24-h dietary survey. A maximum of four adults per household, including the HOH were administered a separate survey. In 2010, women were surveyed on prenatal care and birth, medical, and occupational histories. In 2011, this included questions on occupational, medical, and smoking histories; and spirometry was performed when feasible. In households with more than four adults, the HOH provided guidance on who to interview. ASGM activities were stratified as excavation, crushing (crushing, grinding, or pounding ore), sifting (“shanking”), washing (or sluicing), amalgamation, burning (or roasting), and owning or managing a mine. Since many participants engaged in multiple mining activities, each participant was surveyed about ever-involvement in each mining activity as well as the main activity performed in the three months preceding the survey.

### 2.3. Sample Collection

Urine and hair biological markers were collected to assess elemental and methylmercury exposure, respectively, from all participants when feasible [5]. Spot urine samples (5–15 mL) were collected from participants mid-morning at the time of the interview, stored at room temperature in Bolgatanga, Ghana, and frozen at −20 °C until analysis in the U.S. Samples were thawed to 4 °C and vortexed prior to analysis. Hair was cut as close to the scalp as possible from the occipital region of the head and placed on a sticky-note and stored in a plastic bag until analysis. Only the 2 cm closest to the scalp was used for analysis. Hair samples were washed once with acetone and twice with deionized water, and dried for Hg analysis [24,30].

Household soil samples were collected in 2010 and 2011from Kejetia and in 2012 from a Bolgatanga refinery. Household surface soil was collected from a common area, designated by the HOH. Each household soil sample is a composite of five subsamples taken from the four corners and center of an approximate 30 cm^2^ area [31]. Samples were collected from the top 1–2 cm of soil into sealed WhirlPak bags. Ore samples were collected from miners within Kejetia from various stages throughout the ASGM process (crushing, grinding, and washing) in 2011. In 2012, soil samples were collected at a gold refinery in the regional urban center Bolgatanga (which processes the gold mined from Kejetia), at zero, three, and six meters from the roasting site, following the same collection protocol as household soil samples. Samples were stored at room temperature in Bolgatanga, Ghana at 4 °C until analysis in the U.S. All samples were dried at 110 °C for 16 h and soil sifted through a 2 mm polymer sieve to remove any detritus or stones.

In 2010, fish and sediment samples were collected from around Kejetia. Fish were caught from the adjacent stream and nearby reservoir and purchased from vendors in Kejetia. Samples were whole fish, but were not identified by species names. All fish samples were dried in Bolgatanga and stored at 4 °C until analysis in the U.S. Samples were dried again at 60 °C for 16 h in the laboratory. Whole fish were ground up for analyses to better reflect consumption habits in the Upper East Region.

### 2.4. Sample Mercury Analysis

Total Hg was measured using a Direct Mercury Analyzer-80 (DMA-80; Milestone, Inc., Shelton, CT, USA), following U.S. Environmental Protection Agency (EPA) Method 7473 [32]. Certified reference materials (CRMs; urine: QMEQAS, Institut National de Santa Publique Quebec; hair: National Institute for Environmental Studies Japan; dogfish liver: DOLT-4, National Research Council Canada; soil: San Joaquin Soil 2709, U.S. National Institute of Standards and Technology) were analyzed approximately every ten samples, blanks every five samples and sample replicates at least every nine samples. All soil and sediment samples were run in duplicate and blanks every two samples (four replicates).

The average recovery of CRMs was 91%–92% for NIES hair and 94%–107% for DOLT-4, and 97%–98% for QMEQAS urine in urine, hair, soil, sediment, ore, and fish sample analyses. For San Joaquin soil CRM, the average recovery ranged from 85% to 101% in Gorogo and Kejetia, respectively. In Kejetia and Gorogo samples, within-day variation was <5% for NIES hair and QMEQAS urine, <10% for DOLT-4, and <20% for San Joaquin soil. The average within-day variation of replicates of participants’ samples was low for hair (<6%) and urine (<8.3%). Soil and sediment samples, analyzed in duplicate due to expected higher variation, had an average within-day variation of 6.7%–12.3%.

The average theoretical Hg detection limit (TMDL; 3 × standard deviation of the ng Hg of blanks + average ng Hg of blanks) for hair was 0.058 ng Hg in Kejetia in 2010, 0.126 ng Hg in Kejetia in 2011, and 0.044 ng Hg in Gorogo. No hair samples were below the TMDL. The average TMDL for urine was 0.090 ng Hg for Kejetia in 2010 (1 sample < TMDL), 0.136 ng Hg for Kejetia in 2011 (3 samples < TMDL), and 0.046 ng Hg for Gorogo (21 samples < TMDL). The average TMDL for household soil was 1.853 ng Hg for Kejetia in 2010, 3.030 ng Hg for Kejetia in 2011, and 0.083 ng Hg for Gorogo. The TMDL for Bolgatanga soil refinery samples was 3.987 ng Hg. The TMDL was high for ore samples (3.183 ng Hg), with 3 samples below the TMDL. Sediment samples also had a high TMDL of 1.853 ng Hg, as they were analyzed with household soil samples (11 samples < TMDL). No fish samples were below the TMDL (0.134 ng Hg).

Specific gravity (SG) was measured using a pocket refractometer (PAL-10S, Atago U.S.A., Inc., Tokyo, Japan) to adjust urine samples by urinary dilution using the mean urinary specific gravity (1.016) [33,34]. Specific gravity was used to adjust for urinary flow over creatinine, as it is less influenced by ethnicity, age, sex, and diet [35,36,37]. The equation is as follows, where *p* refers to a participant’s personal urinary Hg and urinary SG values, and the average urinary SG is 1.016:

$$SG-adjusted\;urinary\;Hg_{p}=\;\frac{\left(Avg.\;urinary\;SG-1\right)}{\left(Urinary\;SG_{p}-1\right)}\times Urinary\;Hg_{p}$$

### 2.5. Soil and Hg Ingestion

Soil and dust ingestion was assessed with three potential scenarios: inadvertent ingestion of small quantities of soil and dust; occasional, intentional ingestion of soil and dust (pica); and habitual, intentional ingestion of soil and dust (geophagy). Inadvertent soil and dust ingestion occurs from ingestion of house dust and hand-to-mouth and food contact. In rural northern Ghanaian communities, earthen floors, a long dry season, and mining activities may increase soil and dust ingestion for adults and children. Using estimates of ingestion from the U.S. EPA on average ingestion rates based on age for a general U.S. population and estimates from other studies used in Sub-Saharan Africa, scenarios were evaluated using a central tendency (median or mean) or worst-case scenario that was often the estimated 95% upper limit of ingestion. Despite best efforts, however, there have been no known studies to quantify soil or dust ingestion in Sub-Saharan Africa for non-geophagic adults or geophagic practices in northeast Ghana where our study was based. As such, we used existing estimates of soil and dust ingestion and the relevant calculations. A variety of scenarios were explored. The U.S. EPA estimates soil and dust ingestion for inadvertent ingestion, whereas estimates for pica and geophagy principally refer only to soil ingestion [38].

Mercury ingestion from soil and dust was calculated per day by multiplying the household soil total Hg concentration (note, we did not speciate the soil Hg) with the quantity of soil and dust ingested per day estimated for various scenarios. For all scenarios, the estimated quantity of soil and dust ingested is equal for all participants, except for the inadvertent ingestion central tendency of 50 mg/day for adults ≥21 years and 110 mg/day for adults 18 to 21 years [38]. Lacking a reference dose (RfD) for elemental Hg (which is likely to be the main form of Hg in the soil), Hg ingestion was evaluated with the U.S. EPA RfD for mercuric chloride of 0.3 µg Hg per kg of body weight-day and using participants’ body weight [39]. To assess potential exposure for Bolgatanga residents living near the urban refinery, a set weight of 65 kg was used to determine an RfD. Although the average U.S. adult weight is 80 kg (>21 years) [38], the average weight was 63.2 kg in Kejetia and 59.1 kg in Gorogo. Furthermore, we assumed 100% bioavailability of Hg following ingestion, and without Hg speciation data we used total soil Hg to compare to the RfD. Future studies should further explore these particular issues.

### 2.6. Statistical and Spatial Analyses

The data were analyzed using SPSS Statistical Software (v.22; IBM, Chicago, IL, USA). Since Hg biomarkers and household soil were not normally distributed, they were log-transformed for independent *t*-tests. Bivariate analyses were also performed non-parametrically (e.g., Spearman’s ρ). One-way analysis of variance (ANOVA) was used to assess bivariate correlations of Hg biomarkers and household soil with mining status, mining activities, sex, and education level. Correlations between ever-involvement in each mining activity were assessed through Chi-square tests. Statistical analyses were performed with specific gravity-adjusted and unadjusted urinary Hg. All results are reported with mean ± standard deviation, unless otherwise indicated.

Maps of households in each community were created using ArcGIS (v. 10.1; Esri, Redlands, CA, USA). GPS coordinates were measured at each participant’s household. We were unable to geocode three households due to logistical issues. These three households were exempted from geospatial analyses. This included two households from Kejetia and one from Gorogo. Global Moran’s I statistic was used to analyze spatial autocorrelation of Hg concentrations across the Kejetia community in urine and household soil. In each assessment, Euclidean distance and inverse distance weighting were used to account for Kejetia’s non-grid organization and to place a larger influence on nearby neighbor Hg concentrations than more distant Hg concentrations.

## 3. Results

### 3.1. Demographics and Mining

In 2010, we surveyed 90 women of childbearing age (15–48 years) from 57 households in Kejetia. The women had a mean age of 26.6 ± 7.9 years. Forty-four percent of the participants had received no formal education when the survey was conducted. Thirty-two (36%) of the women were involved with ASGM and 11 (34%) of these women worked directly with Hg-gold amalgams. The most common main mining activity, however, was sifting ore (75%), followed by grinding ore (19%).

There were 97 participants from 54 households in Kejetia and 75 participants from 26 households in Gorogo in 2011 (Table 1). One Gorogo current miner was excluded from analyses. Kejetia participants, on average, were younger (mean: 31.4 ± 10.8 years) and more male (52%) than Gorogo participants (51.5 ± 18.8 years and 45% male). Gorogo participants were largely farmers (95%), while 73% of Kejetia participants were current miners. Smoking rates and cigarette pack-years were low, particularly among females. Education rates were low in both communities, with 39% and 81% of participants in Kejetia and Gorogo, respectively, reporting either only obtaining some preschool or no schooling.

Seventy-seven percent of Kejetia miners had engaged in mining at any time previously (Table S1). The most common mining activity was amalgamation (66%), and owning or managing a mine the least common (28%). Mining activities were performed differentially by sex. Excavation, crushing, washing, and amalgamation were common among males (>60%), while only sifting ore was common among the majority of females (64%). Many miners engaged in multiple mining activities within a workday. Among miners, ever amalgamation was significantly correlated to ever excavation, crushing, washing, and burning (*p* < 0.05); and ever burning was significantly associated to ever excavation, washing, and amalgamation.

**Table 1:** Demographic information of Kejetia and Gorogo participants.

	Kejetia			Gorogo
	All	Miners ^a^	Non-miners ^a^	All
*n* participants	97	71	26	75
*n* households	54	41	18	26
Sex (% Male)	50 (51.5%)	43 (60.6%)	7 (26.9%)	34 (45.3%)
Age (Mean [SD])	31.4 (10.8)	30.6 (9.6)	33.8 (13.6)	51.5 (18.8)
BMI (Mean [SD])	22.7 (3.2)	22.1 (2.7)	24.5 (3.7)	21.8 (3.1) **^b^**
Occupation				
Current Miner	71 (73.2%)	-	-	0 (0%)
Ex-Miner	4 (4.2%)	0 (0%)	4 (15.4%)	10 (13.3%)
Farmer	7 (7.3%)	5 (7.0%)	2 (7.7%)	71 (94.7%)
Cook (food, pito) **^c^**	15 (15.6%)	5 (7.0%)	10 (38.5%)	3 (4.0%)
Vendor	18 (18.8%)	7 (9.9%)	11 (42.3%)	7 (9.3%)
Other	12 (12.5%)	6 (8.5%)	6 (23.1%)	7 (9.3%)
Smoking				
Smoking in home	45 (46.9%)	39 (54.9%)	6 (23.1%)	39 (52.0%)
Current smoker	15 (15.6%)	14 (19.7%)	1 (3.8%)	14 (18.7%)
Ex-smoker	7 (7.3%)	6 (8.5%)	1 (3.8%)	9 (12.0%)
*n* ever-smokers with pack-years **^d^**	16	15	1	14
Cigarette pack-years **^d^**	15.8 (26.6)	15.1 (27.4)	25.5	3.9 (2.1)
Education				
No school	28 (29.2%)	16 (22.9%)	12 (46.2%)	52 (69.3%)
Nursery/preschool	9 (9.4%)	6 (8.6%)	3 (11.5%)	9 (12.0%)
Primary	27(28.1%)	24 (34.3%)	3 (11.5%)	6 (8.0%)
Middle/JSS	20 (20.8%)	18 (25.7%)	2 (7.7%)	1 (1.3%)
Secondary/SSS, tech.	11 (11.5%)	5 (7.1%)	6 (23.1%)	5 (6.7%)
Higher than secondary	1 (1.0%)	1 (1.4%)	0 (0%)	2 (2.7%)

**^a^** Refers to current and non-current miners; **^b^** *n =* 74 for Gorogo: All; **^c^** Includes individuals that cook food or pito, an alcoholic beverage made from millet; **^d^** Cigarette pack-years only include ever-smokers.

### 3.2. Mercury in Biological Samples

**Figure 3:**
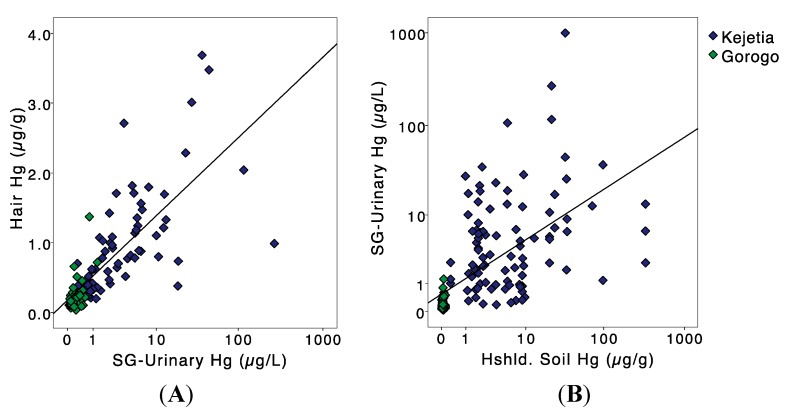
(**A**) Personal specific gravity (SG) adjusted urinary Hg (log-scale axis) by hair Hg concentrations; *R^2^* = 0.592; and (**B**) household soil Hg by personal specific gravity (SG) adjusted urinary Hg concentrations (log-scale axes); *R^2^* = 0.373.

There were significant differences in mean biomarker Hg concentrations between Kejetia and Gorogo in 2011 (Table 2). Kejetia participants had significantly higher mean unadjusted and SG-adjusted urinary Hg (30.9 µg/L and 22.8 µg/L, respectively) than Gorogo participants (0.161 µg/L and 0.216 µg/L, respectively). Mean urinary Hg concentrations were lower among women of childbearing age participants from 2010 (7.82 µg/L) than in 2011 (Table 3). Mean hair Hg was more similar in the two communities, but was still significantly lower in Gorogo (0.231 µg/g) compared to Kejetia (0.974 µg/g). In 2010, mean hair Hg was slightly higher (1.66 µg/g), driven by one outlier (92.6 µg/g). Hair and urinary Hg collected in 2011 displayed a positive relationship; *R^2^* = 0.592 (Figure 3A). In bivariate analyses, SG-urinary Hg was significantly correlated to hair Hg in Kejetia (Spearman’s ρ = 0.765, *p <* 0.001) and Gorogo (ρ = 0.405, *p =* 0.017).

**Figure 4:**
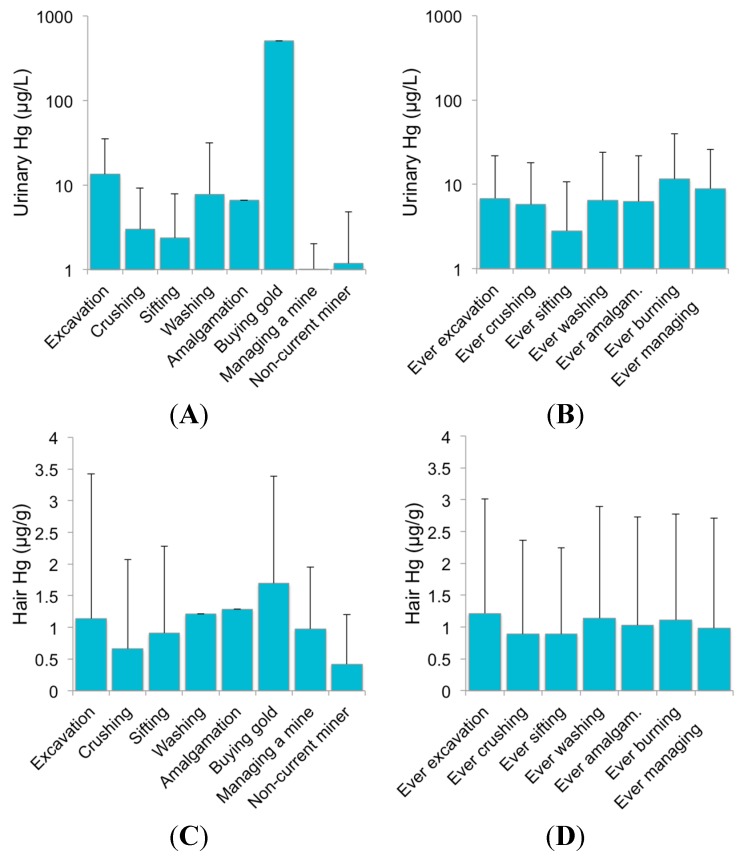
Median urine (**A**, **B**) and hair (**C**, **D**) Hg concentrations by Kejetia participants’ mining involvement. Bars represent mean values with the 75th percentile indicated as error bars. Median values are stratified by the main mining activity performed in the preceding three months of the interview in (**A**) and (**C**); and ever involvement in each mining activity in (**B**) and (**D**).

Figure 4 displays the median Hg concentrations in urine and hair for Kejetia 2011 participants, stratified by their main mining activity from the preceding three months of the interview and ever involvement. Only 2 participants engaged in amalgamation, buying gold, or managing a mine as their main activity, while ever involvement in amalgamation, burning, or managing a mine were more common (*n =* 49, 30, and 19, respectively). Urinary Hg was significantly correlated to ever involvement in excavation, washing, amalgamation, burning, and owning a mine in Kejetia.

Kejetia 2011 current miners had significantly higher mean unadjusted and SG-adjusted urinary Hg, and hair Hg (39.5 µg/L, 29.3 µg/L, and 1.13 µg/g, respectively) than Kejetia non-miners (6.61 µg/L, 4.22 µg/L, and 0.558 µg/g, respectively). Those reporting ever-using Hg in amalgamation or burning in Kejetia had significantly higher unadjusted and SG-adjusted urinary Hg and hair Hg (*n =* 54; 49.8 ± 194 µg/L, 36.6 ± 141 µg/L, and 1.27 ± 0.858 µg/g, respectively) than never-Hg users (*n =* 43; 5.27 ± 10.6 µg/L, 3.92 ± 5.76 µg/L, and 0.627 ± 0.370 µg/g, respectively). Kejetia males had significantly higher mean unadjusted and SG-adjusted urinary Hg and hair Hg (*n =* 34; 54.6 ± 204 µg/L, 39.8 ± 148 µg/L, and 1.24 ± 0.858 µg/g, respectively) than Kejetia females (*n =* 41; 5.03 ± 10.5 µg/L, 4.21 ± 7.44 µg/L, and 0.799 ± 0.615 µg/g, respectively). In a logistic regression of Kejetia participants, males had significantly higher odds of urinary Hg > 10 µg/L (7.42 95% CI: 2.13, 25.8) while adjusting for age, mining status (current or ex), and household soil Hg concentrations.

**Table 2:** Biomarkers and household soil Hg concentrations for all participants.

Biomarker		Kejetia			Gorogo
		All	Miners ^a^	Non-miners ^a^	All
**Urine**	*n*	92	68	24	70
Urinary Specific Gravity (SG)					
	Mean (SD)	1.018 (0.007) **^d^**	1.017 (0.007)	1.020 (0.006)	1.014 (0.006)
Urinary Hg (µg/L)					
	Mean (SD)	30.9 (148.5) **^d^**	39.5 (172.1) **^e^**	6.61 (13.2)	0.161 (0.131)
	Median	2.94	4.83	1.41	0.114
	IQR **^b^**	1.04, 11.0	1.26, 12.9	0.742, 5.23	0.079, 0.217
	Min-Max	0.160–1372	0.160–1372	0.199–58.1	0.026–0.580
	>10 µg/L Hg (%)	25 (27.2%)	21 (30.9%)	4 (16.7%)	0 (0%)
	>50 µg/L Hg (%)	8 (8.7%)	7 (10.3%)	1 (4.2%)	0 (0%)
SG-adj. Urinary Hg **^c^** (µg/L)					
	Mean (SD)	22.8 (107.8) **^d^**	29.3 (124.9) **^e^**	4.22 (6.88)	0.216 (0.194)
	Median	3.35	5.18	1.18	0.154
	IQR **^b^**	1.14, 10.5	1.92, 12.7	0.733, 3.61	0.095, 0.261
	Min-Max	0.18–998	0.188–998	0.212–25.8	0.042–1.24
	>10 µg/L Hg (%)	24 (26.0%)	20 (29.4%)	4 (16.7%)	0 (0%)
	>50 µg/L Hg (%)	4 (4.3%)	4 (5.9%)	0 (0%)	0 (0%)
**Hair Hg** (µg/g)	*n*	70	51	19	59
	Mean (SD)	0.974 (0.748) **^d^**	1.13 (0.809) **^e^**	0.558 (0.272)	0.231 (0.202)
	Median	0.783	0.967	0.419	0.181
	IQR **^b^**	0.408, 1.22	0.589, 1.47	0.329, 0.781	0.119, 0.244
	Min-Max	0.132–3.69	0.132–3.69	0.237–1.10	0.037–1.37

**^a^** Refers to current miners and non-current miners; **^b^** Interquartile range (25th percentile, 75th percentile); **^c^** Specific gravity adjustment equation: Urinary Hg × ((1-avg. SG)/(Urine SG-1)); **^d^** Gorogo *vs.* Kejetia *t*-test comparing means of log-transformed data (except for specific gravity), *p* < 0.001; **^e^** Miners *vs.* non-miners *t*-test comparing means of log-transformed data, *p* < 0.05.

**Table 3:** Total mercury (Hg) concentrations (µg/g d.w.) in urine (µg/L), hair, soil, sediment, and fish samples collected 2010–2012 in Gorogo, Kejetia, and Bolgatanga, Upper East Region.

Study Location	Media	Year Collected	*n*	Mean (SD)	Median	Min.-Max.	Exceeds Guideline (%)	Guideline Value
Kejetia women of childbearing age	Urine	2010	84	7.82 (38.2)	1.38	0.096–336.7	5 (6.0)	10 µg/L **^a^**
Kejetia women of childbearing age	Hair	2010	80	1.66 (10.3)	0.359	0.097–92.6	1 (1.3)	11.1 µg/g **^b^**
Gorogo households	Soil	2011	26	0.041 (0.023)	0.039	0.013–0.114	0 (0%)	6.6 µg/g **^c^**
Kejetia households	Soil	2010	17	4.78 (9.78)	2.16	0.096–40.969	3 (17.6)	
Kejetia households	Soil	2011	54	15.6 (46.9)	3.05	0.297–330.04	18 (33.3)	
Bolgatanga gold refinery	Soil	2012	4	57.8 (58.2)	54.6	5.43–116.44	3 (75.0)	
Kejetia and surrounding areas	Sediment	2010	14	0.036 (0.062)	0.021	0.005–0.248	1 (7.1)	0.170 µg/g **^d^**
Kejetia and surrounding areas	Fish (unknown fresh water species)	2010	12	0.070 (0.057)	0.045	0.024–0.220	0 (0%)	0.3 µg/g **^e^**

**^a^** Expected to be asymptomatic <10 µg/L [40]; **^b^** U.S. EPA [15] benchmark dose of 11.1 µg/g; UNEP guideline of 10 µg/g [41]; **^c^** Canadian Soil Quality Guideline for inorganic Hg at residential sites [42]; **^d^** Canadian Sediment Quality Guideline for Hg in freshwater sediment [43]; **^e^** U.S. EPA Tissue Residue Criterion for MeHg in fish [44].

**Figure 5:**
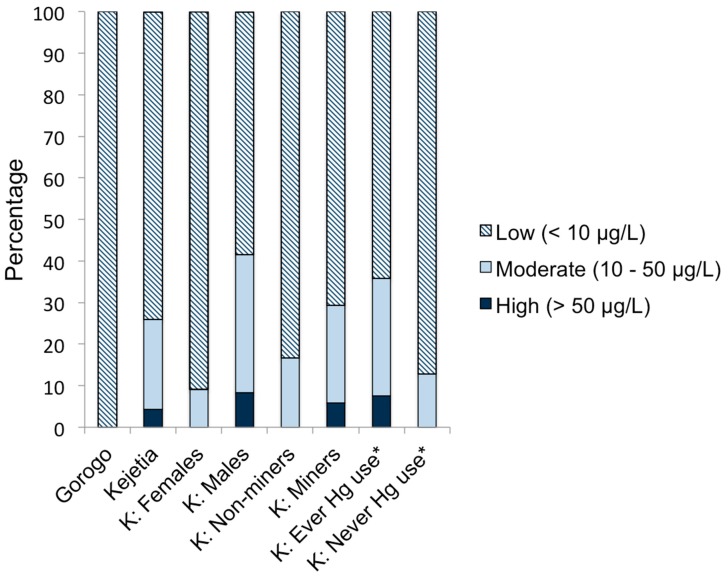
Percentage of participants with low (<10 µg/L), moderate (10–50 µg/L), and high (>50 µg/L) SG-Urinary Hg. * Ever Hg use includes ever involvement in amalgamation or burning.

The World Health Organization (WHO) recommends a health-based limit of 50 µg/L inorganic Hg (creatinine-adjusted) for occupational exposure to protect workers from tremors and Hg-induced non-specific symptoms [5,45]. In Kejetia, 4% of participants from 2011 had SG-adjusted urinary Hg concentrations above this recommended limit (Table 2). Twenty-six percent of 2011 Kejetia participants had moderately high SG-adjusted urinary Hg concentrations (>10 µg/L, the expected threshold for symptoms) [40]. Six percent (*n =* 5) of women of childbearing age sampled in 2010 had urinary Hg concentrations exceeding 10 µg/L. A summary of low, moderate, and high urinary Hg concentrations are displayed in Figure 5. Hair Hg concentrations were all below the U.S. EPA benchmark dose of 11.1 µg/g total hair Hg [15], and high exposures >10.0 µg/g Hg designated by the WHO and UNEP [41], except for one extreme outlier in 2010 (at 92.6 µg/g).

### 3.3. Mercury in Ecological Samples

Mercury concentrations varied in ecological media (Table 3). Mean household soil Hg collected in 2011 in Kejetia (15.6 µg/g) was significantly higher than in Gorogo (0.041 µg/g). Kejetia current miners had significantly higher mean household soil Hg (18.72 µg/g) than Kejetia non-miners (5.546 µg/g). Household soil was higher among Kejetia participants reporting ever-using Hg in amalgamation or burning (*n =* 54; 36.07 ± 85.70 µg/g), compared than never-Hg users (*n =* 43; 7.506 ± 15.90 µg/g). In 2010, Hg levels in household soil samples were lower (*n =* 17; 4.78 ± 9.78 µg/g) than in 2011. A third of all Kejetia households sampled in 2011 and 18% of houses sampled in 2010 had soil Hg concentrations exceeding the Canadian Soil Quality Guideline of 6.6 µg/g inorganic Hg for the protection of human and environmental health at residential sites [42]. Soil Hg concentrations in Gorogo were all below levels of concern. Household soil was significantly correlated to urinary Hg in Kejetia (ρ = 0.238, *p =* 0.023); *R^2^* = 0.373 (Figure 3B). In a logistic regression of Kejetia participants adjusting for age, sex, and mining status, household soil Hg concentration was not a significant predictor of SG-urinary Hg > 10 µg/L.

Sediment and fish Hg concentrations collected around Kejetia were quite low (mean *=* 0.036 µg/g and 0.070 µg/g dry weight, respectively). One sediment sample exceeded the Canadian Sediment Quality Guideline of 0.17 µg/g Hg [43]. Using 80% moisture for the fish samples, the wet weight total Hg concentrations from Kejetia-area fish (mean: 0.014 ± 0.012 µg/g wet weight) were all below the U.S. EPA guideline of 0.3 µg/g for MeHg in fish [44]. Ore samples (*n =* 10) were collected from various stages in the mining process: crushing, milling, and washing (Table S2). Mean concentrations were highest in crushed ore (*n =* 4; mean: 1.49 µg/g) and lowest in washed ore/tailings (*n =* 2; mean: 0.35 µg/g).

Soil samples collected at and around the Bolgatanga gold refinery had very high Hg concentrations. Two samples collected from within the refinery were unable to be analyzed because Hg concentrations were above measurable levels (*i.e.*, 1200 ppm). Three of the four remaining samples exceeded the Canadian Soil Quality Guideline [42].

**Figure 6:**
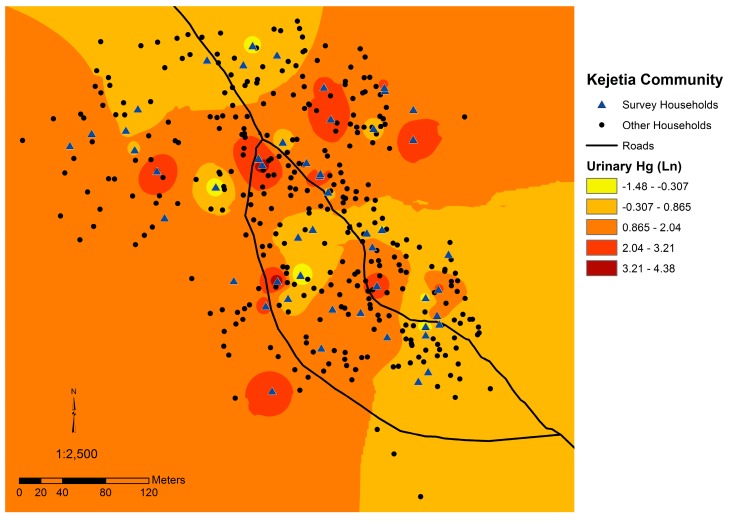
Map of Kejetia including an estimation of mean urinary Hg (log-transformed) throughout the community. Darker red indicates higher estimated urinary Hg concentrations for residents based on household locations.

### 3.4. Spatial Analyses

**Figure 7:**
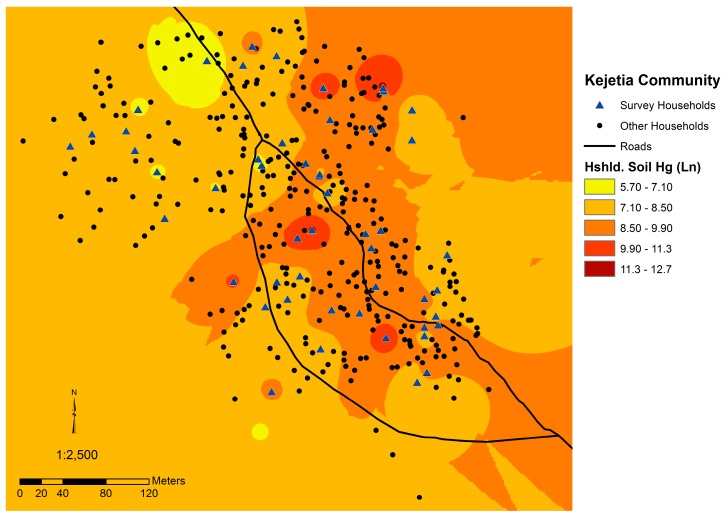
Map of Kejetia including an estimation of mean household soil Hg (log-transformed) throughout the community. Darker red indicates higher estimated household soil Hg concentrations.

Urinary Hg and household soil Hg were both spatially auto-correlated, suggesting that Hg concentrations in each media is not randomly distributed throughout the community. The mean household urinary Hg (log-transformed) was significantly auto-correlated (*p* = 0.007, z-score of 2.67, Moran’s index = 0.2687). Figure 6 displays the urinary Hg concentrations estimated throughout Kejetia from our sampling data. Household soil Hg was also significantly auto-correlated (*p* < 0.001, z-score of 11.28; Moran’s index = 1.179). Figure 7 displays soil Hg concentrations estimated throughout Kejetia from our sampling data.

**Table 4:** Soil and dust ingestion scenarios and Hg ingestion estimations (mean) in Kejetia, Gorogo, and Bolgatanga by ingestion scenario.

		Inadvertent Ingestion		Occasional Ingestion (Pica)		Habitual Ingestion (Geophagy)	
		Central Tendency	Worst-case	Central Tendency	Worst-case	Central Tendency	Worst-case
Population	Reference population:	U.S. adults	US adults; Tanzanians	US adults	Tanzanians	US adults	Tanzanians
	Reference:	[38]	[38,47,48]	[38]	[48]	[38]	[48]
	Soil & dust ingestion (mg/day): **^a^**	50; 110 **^b^**	200	1000	13600	50000	85000
Kejetia	µg Hg ingested/day	2.06	4.68	23.4	318	1170	1990
	µg Hg/kg body weight/day	0.037	0.082	0.409	5.57	20.5	34.8
	*n* (%) > 0.3 µg Hg/kg body weight/day **^c^**	4 (4.1)	6 (6.2)	20 (20.8)	84 (86.6)	94 (96.9)	96 (99.0)
Gorogo	µg Hg ingested/day	0.002	0.008	0.042	0.574	2.11	3.58
	µg Hg/kg body weight/day	3.9 × 10^−5^	2.0 × 10^−4^	8.0 × 10^−4^	0.01	0.038	0.064
	*n* (%) > 0.3 µg Hg/kg body weight/day **^c^**	0 (0%)	0 (0%)	0 (0%)	0 (0%)	0 (0%)	0 (0%)
Bolgatanga Refinery: Adults	µg Hg ingested/day	2.89 **^d^**	11.56	57.8	786.08	2890	4913
	µg Hg/kg body weight/day **^e^**	0.044	0.178	0.889	12.1	44.5	75.6

**^a^** Only inadvertent ingestion explicitly accounts for soil and dust ingestion; other estimates for soil ingestion; **^b^** Ingestion varies by age: <21 years = 110 mg/day; >21 years = 50 mg/day [38]; **^c^** US EPA reference dose (RfD) for mercuric chloride [39]; **^d^** Ingestion rate at 50 mg/day; **^e^** Assigned weight for adults is 65 kg.

### 3.5. Soil and Hg Ingestion

Lacking precise information on soil ingestion in the region, a variety of scenarios were explored here. Estimated quantities of inadvertent, occasional, and habitual ingestion of soil and dust are listed in Table 4 by the central tendency and worst-case (often corresponding to the 95% upper limit) scenarios. While many estimates are from an adult U.S. population, other estimates were considered from Ghana, Tanzania, Nigeria, and Kenya [19,20,23,38,46,47,48]. Using ingestion quantities summarized in Table 4, we calculated the estimated daily total Hg ingestion via soil and dust in each population. In Kejetia, the estimated daily total Hg ingestion from soil and dust ranged from 2.06 to 11.7 µg/g per day in inadvertent ingestion, 23.4 to 318 µg/day in occasional ingestion, and 1170 to 1990 µg/day in habitual ingestion. Inadvertent ingestion of soil at 500 mg/day corresponded to an estimated nine Kejetia participants exceeding the U.S. EPA reference dose (RfD) of 0.3 µg Hg/kg body weight/day for mercuric chloride [39]. Almost all Kejetia participants would exceed the RfD under geophagic ingestion scenarios, and 21% and 87% of pica soil ingestion at the central and worst-case scenarios, respectively.

The Bolgatanga refinery is located in a residential area within the Bolgatanga Municipal District, with a population of over 148,000 [49]. Hg ingestion was estimated for adults in Bolgatanga using the mean soil Hg concentration from the refinery (Table 4). The average adult weight was estimated to be 65 kg, determined as a an estimate from the mean weight in Kejetia (63.2 kg) and Gorogo (59.2 kg), and the U.S. average adult weight of 80 kg [38]. Inadvertent ingestion at 65 kg body weight would not be above the RfD, but ingestion of greater than 200 mg of soil per day would likely result in exposures above the RfD.

## 4. Discussion

In Kejetia, like other ASGM communities, Hg contamination is of prominent concern. Our study assessed the Hg contamination of soil, sediment, and fish, and human exposure through multiple biomarker measures. Mining involvement and use of Hg was common in Kejetia—just over 50% of Kejetia 2011 participants performed amalgamation, indicating a potential for widespread contamination and exposures. Urinary and hair Hg biomarkers, which were positively correlated, were elevated among Kejetia and Gorogo participants.

Studies of other ASGM communities generally had higher mean hair Hg concentrations than in Kejetia (0.974 ± 0.748 µg/g), but this varied (means ranging from 0.62 to 4.27 µg/g) [50,51]. Kejetia miners (1.13 ± 0.809 µg/g) had similar hair Hg concentrations to Kejetia miners surveyed in 2009 (1.2 ± 1.4 µg/g) [24] and to other ASG miners across Ghana (means ranging from 1.1 to 2.14 µg/g) [24,51,52]. Most participants had hair Hg concentrations between 1 and 2 µg/g, a “normal level” [41]. Mean hair Hg concentrations were lower in Gorogo (0.231 ± 0.202 µg/g) than observed in other non-mining populations in Ghana (means ranging from 0.717 to 2.35 µg/g) [51,52,53,54]. Other studies of non-miners have included more urban participants that may have a higher diet of fish contributing to higher hair Hg concentrations. In addition, our analysis of Hg in sediment and fish found that concentrations were low, as seen across Ghana [7], which may partially explain our low hair Hg levels.

External contamination is a major challenge in using hair as a biomarker for MeHg in ASGM communities. Sherman *et al.* [14] examined Hg stable isotopes in urine and hair to assess their validity as biomarkers for elemental and MeHg. The study used hair and urinary Hg from Kejetia miners collected in our 2010 and 2011 study. Total urinary Hg had Hg isotope ratios similar to those found in ore deposits, and accurately reflect exposure to inorganic elemental Hg used in ASGM. Hair Hg, however, had a low percentage of MeHg as total Hg (7.6–29%), suggesting that the majority of hair Hg was exogenously adsorbed elemental Hg [14]. It is likely that hair Hg overestimates exposure to MeHg from fish and seafood consumption in these ASGM communities [14]. The one outlier from 2010 (92.6 µg/g hair Hg) may be an example of high exogenously adsorbed elemental Hg on hair. Our hair samples were washed with acetone and deionized water, but other studies have found that these procedures do not remove adsorbed, external Hg contamination [17]. It is possible to analyze hair samples for MeHg specifically, but this requires a larger mass of hair, and sample quantities are often limited as many Ghanaians had hair <2 cm. Thus, hair samples from Kejetia and ASGM sites likely do not represent merely MeHg as is often found in populations not exposed to elemental or inorganic Hg.

Kejetia 2011 participants had higher mean urinary Hg concentrations (30.9 µg/L) than observed in most other studies of ASGM communities in southern Ghana (means ranging from 2.6 to 34.2 µg/L) [50,55]. Slight differences in the ASGM process and practices, such as communities built solely around ASGM, may lead to higher exposures in the Upper East Region than in southern Ghana where people may not live as close to mining activities. Kejetia miners from our study had similar, but slightly elevated, urinary Hg levels from a 2009 study of Kejetia miners (mean: 38.9 ± 95.7 µg/L) [24], and other studies of ASGM miners in Ghana (means ranging from 0.56 to 17.0 µg/L) [24,52,55,56]. Mean urinary Hg in Gorogo (0.161 µg/L) was lower than observed in other non-ASGM communities (means ranging from 0.69 to 3.1 µg/L) [52,55].

Mercury is a known toxicant with numerous adverse health effects. Hg is especially dangerous as it is able to pass the blood-brain and blood-placental barrier, posing additional risks to an unborn fetus and young children [5]. Evidence in Kejetia and elsewhere show that its use is common in ASGM communities, posing potential risks for miners and community residents. While <10 µg/L Hg is expected to be asymptomatic [40], women of childbearing age are recommended to be exposed to as little Hg as possible [5]. The 24 Kejetia 2011 participants with SG-adjusted urinary Hg concentrations >10 µg/L may be at risk for Hg-induced toxicity [5,45]. Almost 17% of non-mining participants in Kejetia had urinary Hg concentrations >10 µg/L, suggesting that living within the community exposes residents to Hg vapor, even without engaging in mining directly. This may result from inhalation of directly burned elemental Hg or inhalation of volatilized soil Hg. This is particularly important for pregnant women and young children that also reside in these communities and are more vulnerable to the impacts of Hg exposure [5].

Household soil Hg concentrations were significantly higher in Kejetia than Gorogo. Household soil Hg was spatially autocorrelated and positively correlated to whether Kejetia participants were current miners and if they had ever burned Hg. Soil Hg in Kejetia (medians in 2010 and 2011, respectively: 2.16 and 3.05 µg/g; range: 0.096–330 µg/g), were similar to soil Hg levels from two ASGM villages in Zamfara, Nigeria (medians: 2.4 and 2.9 µg/g; range: 0.7–15.2 µg/g) [19]. Mean Kejetia household soil Hg (15.55 µg/g) in 2011 was higher than measured in studies of 19 other ASGM areas across Ghana except for one study of four sites in the Western and Central Regions, which measured soil from active amalgamation and burning sites and found mean soil Hg levels of 0.93, 6.09, 21.6, and 185.93 µg/g [7,57]. Our study, however, focused on common areas where people spent their time to more appropriately reflect personal exposures for community residents. In other studies across Ghana, 21 out of 25 (84%) ASGM sampling sites measured mean soil levels below the Canadian Soil Quality Guideline for residential areas at 6.6 µg/g Hg, and 3 out of 25 (12%) were below 0.1 µg/g Hg [7,51,57,58,59,60,61,62,63,64,65]. Household soil Hg in Gorogo (0.041 ± 0.023 µg/g) was lower than observed in other non-ASGM areas (means ranging from 0.099 to 0.170 µg/g) [7,51,64].

High household soil Hg concentrations exceeding the Canadian Soil Quality Guideline for residential areas, which is set to protect human and environmental health, indicate an additional concern [42]. One-third of 2011 Kejetia household soil Hg concentrations were above the Canadian Soil Quality Guideline of 6.6 µg/g Hg [42,45]. Kejetia household soil Hg was significantly correlated to urinary Hg in 2011, suggesting that Hg burning is occurring at or around participants’ homes and may be locally depositing in the community. The spatial autocorrelation of household soil and participants’ urinary Hg in Kejetia further supports this hypothesis. The correlation of urinary Hg and household soil may also be in indication of inhalation of evaporated elemental Hg from contaminated household soil. As seen in Figure 2, mining activities occur throughout the community, which may explain why contamination and exposures are widespread in Kejetia.

Mean and median soil Hg concentrations from samples collected at the Bolgatanga refinery were higher than at Kejetia, Zamfara (Nigeria), and most other studies of ASGM sites in Ghana [7,19]. This is of concern given that such refineries are common across countries with active ASGM industries and are often situated outside the ASGM community in larger urban centers where few have explored Hg exposure issues. Concentrations of Hg from ore samples were relatively low compared to soil samples, but were greater than measured in Gorogo or other non-ASGM sites across Ghana [7]. Sediment and fish samples were quite low, and all fell below guideline values except for one sediment sample [43,44].

Ingestion of soil, particularly through the practice of geophagy and pica, may be an additional pathway of exposure to Hg that is perhaps understudied in ASGM communities. The dusty environment in northern Ghana combined with the mining activities may also result in higher inadvertent soil ingestion. People may ingest soil and dust through hand-to-mouth contact, the air, and from soil and dust on skin, clothing, inanimate objects, and food. Although the prevalence of the practice of geophagy in northern Ghana is unknown, Vermeer’s study indicates it was common in the Volta Region in the 1970s (prevalent in 46.4% of adult females), and is still common in many Sub-Saharan African countries [20,21]. Vermeer also found that pica was common in 73.6% of adult females and 13.9% of adult males [21]. The fractionation of Hg in household soil is unknown, however, and total Hg concentrations may not accurate reflect bioavailability.

Any Kejetia adults practicing geophagy and upper limit pica may be at risk of exceeding the U.S. EPA RfD for Hg ingestion, and still others may ingest quantities of Hg greater than the RfD in inadvertent ingestion. Children and youth, who ingest higher quantities of soil and dust and have greater hand-to-mouth contact, may have a larger burden of Hg exposure from soil and dust than adults. In geophagy, soil and clays may be purchased at markets from elsewhere, or be locally sourced from walls of houses, termite mounts, and ground soil [21,23]. Geophagic consumption of local or distant soils and involvement in ASGM would greatly influence Hg exposure. High soil concentrations around the refinery in Bolgatanga stress the important of studying urban areas where gold is refined, in addition to traditional ASGM areas where ore is processed.

As the share of global anthropogenic Hg from ASGM grows, there is increasing worldwide concern about its use. The Minamata Convention on Mercury is a global treaty working to reduce Hg emissions and mining with a particular focus on regulating the ASGM sector [66]. Convention signatories, such as Ghana, are expected to take initiatives to reduce and when feasible eliminate the use of Hg and to develop a national action plan (Article 7, Annex C) including various elements such as reduction targets and baseline estimates of Hg used in ASGM [66]. Studies such as this one provide necessary support for the Minamata Convention’s assessment of baseline Hg use and pollution in ASGM, particularly filling a knowledge gap in northern Ghana, since most studies have focused on southern Ghana.

### Limitations

There are a number of limitations to consider for this study. Data on air Hg concentrations in homes and throughout the community would provide a more complete picture of exposure pathways. High household soil Hg concentrations may be a source of Hg vapor in homes and an additional inhalational exposure. The Canadian guideline values for Hg in soil and sediment are for non-tropical environments and do not include Hg speciation [42,43]. Owing to inconsistent practices, recipes, and meal sharing, and a lack of knowledge of fish species and origins, it is difficult to adequately quantify fish consumption for rural Ghanaians. An estimate of MeHg ingestion from fish consumption would help to better estimate the fraction of MeHg in ASGM community participants’ hair samples. However, utilizing Sherman *et al.*’s study [14], we have a suitable estimation of the proportion of MeHg in ASGM miners’ hair.

Many of the soil and dust ingestion scenarios from US-based assumptions of inadvertent ingestion and pica are inadequate for West Africans with different dietary habits, earthen floor housing, climate, local environments, and other factors [19]. It is unclear what appropriate soil and dust ingestion rates are for rural northern Ghanaians, but the dusty conditions during the long dry season from October through April anecdotally imply higher ingestion rates [67]. ASGM areas may also have higher ingestion rates as people work and live in close proximity to ore processing [48]. Soil ingestion rates from Tanzanian studies reflect only soil ingestion, and may significantly underestimate total soil and dust ingestion in dusty conditions. Estimations of Hg ingestion from soil were based on household soil concentrations, although we do not have information on the source of soil for geophagy. In northeast Ghana and in a mining community in particular, individuals practicing geophagy may also intentionally obtain soils from outside their community, in community spaces, or from their homes.

The toxicity of Hg depends greatly on its speciation, which is unknown for Kejetia. A site contaminated with mercuric nitrate and elemental Hg in Tennessee, USA, measured total Hg from 0.5 to 3000 µg/g in floodplain soil [68]. Only 0.01% was found to be organic Hg (MeHg), 6% was elemental Hg, and 91% was inorganic (primarily HgS) [68]. A follow-up study at the same site 15 years later measured elemental Hg in 10%–30% of total Hg [69]. At both time points, the soluble and bioavailable fractions were relatively small portions of the total Hg [68,69]. The presence of certain types of bacteria in the soil (e.g., sulfate-reducing bacteria), which is largely unknown for Ghana, can also influence the speciation in soil [68]. In the absence of data on Hg speciation and fractionation in Kejetia, we rely on total Hg concentrations and must also include some uncertainty in our analyses. Further studies on the bioavailability of Hg from soil and dust and ingestion rates in rural, West African communities would aid in clarifying this exposure pathway. Additionally, the U.S. EPA RfD is for mercuric chloride and may not adequately assess risks from elemental Hg ingestion.

## 5. Conclusions

This study increases our understanding of Hg exposures among miners and non-miners living in an ASGM community to better explain the distribution of Hg contamination in these types of communities. As observed in other ASGM communities, Hg exposures are elevated in Kejetia for miners and non-miners. Urinary and household soil Hg concentrations were significantly higher for miners in Kejetia, but still elevated for Kejetia non-miners compared to non-mining communities. Political and research foci are often on miners, but our study emphasizes the considerable impacts to populations indirectly involved with ASGM. Soil and dust ingestion, inadvertently or through pica or geophagy, and inhalation of volatilized Hg from soil may represent additional pathways of exposure to Hg in ASGM communities. Our assessment of Hg contamination in multiple media affords a more holistic and integrated understanding of ecological contamination and human exposures. Further, by mapping ASGM activities, household locations, and relating this to Hg exposure biomarkers we are able to show that contamination by Hg is widespread across such communities. While urban, non-mining populations are generally perceived as having little Hg exposure, elevated soil Hg concentrations from the Bolgatanga refinery stress the need to better understand the exposure for the surrounding populations.

## Supplementary Materials

**Table S1:** Ever mining involvement for Kejetia participants. Participants were surveyed on ever-engaging in each mining activity.

Mining Activity	Kejetia		Miners ^a^		Females		Males	
	*n*	Percent	*n*	Percent	*n*	Percent	*n*	Percent
Any mining activity	75	77.3	71	100.0	31	66.0	44	88.0
Excavation	40	41.2	39	54.9	2	4.3	38	76.0
Crushing	45	46.4	44	62.0	7	14.9	38	76.0
Sifting	45	46.4	42	59.2	30	63.8	15	30.0
Washing	46	47.4	45	63.4	13	27.7	33	66.0
Amalgamation	49	50.5	47	66.2	16	34.0	33	66.0
Burning	31	32.0	31	43.7	7	14.9	24	48.0
Owning	21	21.6	20	28.2	1	2.1	20	40.0

**^a^** Refers to current miners only.

**Table S2:** Kejetia ore Hg concentrations.

Ore Mining Stage	*n* Samples in Ore Stage	Mean (SD) (µg/g Hg)
Crushed	4	1.486 (0.933)
Finely ground	4	0.9263 (0.7609)
Washed	2	0.3492 (0.1006)
